# Dynamic cerebral autoregulation is an independent outcome predictor of acute ischemic stroke after endovascular therapy

**DOI:** 10.1186/s12883-020-01737-w

**Published:** 2020-05-15

**Authors:** Ge Tian, Zhong Ji, Kaibin Huang, Zhenzhou Lin, Suyue Pan, Yongming Wu

**Affiliations:** Department of Neurology, Nanfang Hospital, Southern Medical University, Guangzhou, China

**Keywords:** Dynamic cerebral autoregulation, Risk factors, Acute ischemic stroke, Endovascular therapy, Outcome, Predictor

## Abstract

**Background:**

Endovascular therapy (EVT) is increasingly used to improve cerebral reperfusion after moderate-to-severe acute ischemic stroke (AIS). However, the influence of hemodynamic factors on clinical outcome is still unclear after EVT. Dynamic cerebral autoregulation (dCA) is an important brain reserve mechanism and is impaired after AIS. This study aimed to explore the role of dCA in predicting the outcome of AIS patients after EVT.

**Methods:**

AIS patients with severe stenosis/occlusion of unilateral middle cerebral artery (MCA) or internal carotid and treatment with EVT were enrolled to receive dCA examinations at the 24 h, 72 h and 7th day after stroke onset. Healthy volunteers were also recruited as controls. DCA was recorded from spontaneous fluctuations of blood pressure and MCA flow velocity. Transfer function analysis was used to derive dCA parameters, including phase difference (PD) and coherence in the low-frequency range (0.06–0.12 Hz). The clinical outcome was measured using the modified Rankin Scale (mRS) at 90 days after onset. Multivariate logistic regression was performed to reveal the correlation between dCA and clinical outcomes. The receiver operation characteristics (ROC) curve was performed to determine the cut-off point of PD.

**Results:**

A total of 62 AIS patients and 77 healthy controls were included. Compared with controls, dCA were impaired bilaterally till to 7th day after onset in patients, presenting as much lower PD value on the ipsilateral side. During follow-up, we found that PD on the ipsilateral side at 24 h after onset was significantly lower in patients with unfavourable outcome (*n* = 41) than those with favourable outcome (*n* = 21), even after adjustment of confounding factors (*p* = 0.009). ROC curve analysis revealed that PD < 26.93° was an independent predictor of unfavourable-outcome.

**Conclusion:**

In AIS patients after EVT, dCA was impaired on both sides over the first 7 days. PD on the ipsilateral side at 24 h after onset is an independent unfavourable-outcome predictor for AIS after EVT.

## Background

Acute ischemic stroke (AIS) is a leading cause of death and adult disability worldwide [1]. In Asian populations, large intracranial artery occlusive disease is the most common cause of ischemic stroke [2, 3]. Endovascular therapy (EVT) has been repeatedly proved to improve cerebral reperfusion for severe AIS caused by the occlusion of large arteries in several randomized clinical trials since 2015 [4–8]. EVT can achieve a complete revascularization ratio of 67–88% [4–8]. However, about 56% of patients remain to experience poor outcome even after endovascular treatment [9]. The causes for the lack of improvement in those cases remain incompletely understood [10].

Dynamic cerebral autoregulation (dCA) is a pivotal mechanism to maintain stable cerebral hemodynamics [11]. It is regarded as an intrinsic protective mechanism of the brain, which ensures relatively constant cerebral blood flow (CBF) despite fluctuations in arterial blood pressure (ABP) or cerebral perfusion pressure [12]. However, dCA may be impaired or even vanish after ischemic stroke [13]. That means under pathological conditions that dCA was dysfunctional, CBF tends to passively vary with changes in ABP, leading to brain edema, intracranial hypertension, and consequential deterioration of neurological functions and clinical outcomes [14]. Since EVT mainly changes cerebral hemodynamics in AIS, we speculated that the integrity of dCA might affect the prognosis of patients after EVT through regulating cerebral hemodynamics. If the correlation between dCA and outcome is established, dCA may be used as an early hemodynamic marker to guide early management in patients who received EVT.

In this study, we sought to assess the dysfunctional pattern of dCA in AIS patients who received EVT. Then we aimed to explore the correlation between dCA and outcome for AIS patients treated with EVT, to find out whether that dCA is an independent outcome predictor and to determine the cut-off point to provide a novel prognostic tool.

## Methods

### Participants

The study was approved by the Medical Ethics Committee of Nanfang Hospital. Written informed consent was obtained from all participants. During October 2017 to February 2019, AIS patients were recruited if they: (1) aged between 18 and 85 years; (2) had a baseline National Institutes of Health Stroke Scale (NIHSS) scores of 4 to 24; (3) had an acute, unilateral side severe stenosis/occlusion of middle cerebral artery (MCA) or internal carotid; (4) underwent EVT within 24 h after the onset of symptoms [15]; (5) had a Thrombolysis In Cerebral Infarction (TICI) score [16] of ≥2b after EVT; (6) had a sufficient bilateral temporal bone window for insonation of MCA. Patients who were diagnosed as cancer or mental diseases were excluded. Patients received intravenous t-PA treatment before EVT were also regarded as eligible if the t-PA treatment was in a standard dose (0.9 mg/kg body weight) and given with 10% as a bolus and the remainder infused over 1 h (maximum dose, 90 mg). EVT decisions were based on the NIHSS score, occlusion site according to magnetic resonance angiography (MRA), and Arterial Spin Labeling (ASL) mismatch. EVT was performed by interventional neurologists using Solitaire (Solitaire, Covidien/ev3, Dublin, Ireland; FR revascularization device). The angiographic procedure had to begin and be completed within 24 h after the onset of stroke. If the patient had not received intravenous tPA, heparin infusion was started intravenously with a 2000-unit bolus, followed by an infusion of 450 units per hour during EVT, and was discontinued at the end of the procedure [17, 18]. We set up a healthy volunteers’ database who attended the annual physical examination in Nanfang Hospital from March 2017 to May 2017. In this study, we recruited healthy controls who were age-matched with AIS patients from the healthy volunteers’ database. They were without cerebrovascular risk factors and also should meet the following inclusion criteria: (1) without intracranial and extracranial vascular stenosis by transcranial Doppler sonography (TCD), carotid artery color Doppler (CD) examination; (2) had a sufficient bilateral temporal bone window for insonation of MCAs; (3) the absence of atrial fibrillation, hyperlipidemia, hypertension, diabetes mellitus and cerebral vascular disease history; (4) without a history of chronic physical or mental diseases, without an infectious disease in the past month, without a history of smoking or heavy drinking, no being pregnant or lactating.

The clinical prognosis was assessed with the modified Rankin Scale (mRS) [19], with mRS of 2 or less defined as a favourable outcome at 90 days. All mRS assessments at 90 days after stroke onset were performed by two investigators who were unaware of the study protocol.

### Dynamic cerebral autoregulation (dCA) protocol

The dCA examination protocol was performed according to the white paper from the International Cerebral Autoregulation Research Network [20]. All healthy control subjects were asked to avoid nicotine, caffeine, alcohol, and all kinds of sleep medicines for at least 24 h before the dCA examination. The examination was performed bedside with minimal surrounding stimuli. The control subjects and the patients rested in a supine position with uncrossed legs for more than 15 min before the examination. First, the baseline arterial blood pressure (ABP) was measured at the brachial artery using an automatic blood pressure monitor (Omron 711). Second, we simultaneously recorded continuous spontaneous ABP via a servo-controlled plethysmograph placed around the left middle finger held at the level of the heart (Finometer Pro, Netherlands) and continuous MCA blood flow velocity (BFV) at a depth of 45 mm to 60 mm with 2 MHz probes attached to a customized head frame (EMS-9 PB, Shenzhen, China). Meanwhile, the PaCO_2_ level was also monitored, maintaining in stable rang. Data were recorded for 15 min for further data examination analysis. The artifacts were manually removed after recording.

The dCA analysis was performed using the multimodal real-time analysis software ICM+ invented by Brain Physics Lab of Cambridge University. According to the continuous ABP signal and bilateral MCA blood flow recordings, autoregulation indices including phase difference (PD) and coherence between the two signal components in the specific frequency domain range (0.06–0.12 Hz) was calculated by the transfer function. We used coherence as a data quality control parameter, and only when the coherence was greater than 0.4, the data were included in the subsequent statistical analysis.

### Statistical evaluation

Continuous variables with normal distribution were presented as mean ± standard deviation, and non-normally distributed continuous variables were presented as median (interquartile range, IQR). Kolmogorov–Smirnov analysis was used to test the normality of data distribution. Frequencies (percentages) were measured for categorical variables. Dynamic CA data of healthy controls were analyzed based on the mean value of the left and right cerebral sides. To assess intergroup differences, we used Student’s t-test (normally distributed), Wilcoxon test (not normally distributed) and Chi-square or Fisher’s exact test as appropriate. To determine the relationship between studied variables, analyses of correlation was used. A multivariate logistic regression model was constructed. We derived crude and adjusted odds ratios of the magnitude of PD and an unfavourable clinical outcome from logistic regression. Odds ratios were adjusted for confounding variables, which were different between the 2 subgroups in univariate analysis (*p* < 0.1), including Fast blood glucose, triglyceride (TG), fast blood glue, body mass index (BMI), C-reactive protein (CRP), and NIHSS on admission. *P* values < 0.05 were considered statistically significant. The receiver operation characteristics (ROC) curve was used to get the cutoff point of PD to predict the unfavourable outcome after EVT. All statistical calculations were performed using SPSS 19 (SPSS, Chicago, IL, USA).

## Results

### Participant characteristics

In 71 AIS patients, 2 cases were EVT failure, 2 cases were diagnosed as cancer, and 5 cases refused to receive EVT. A total of 62 AIS patients (55.6 ± 14.5 years; 73% males) who underwent EVT were enrolled in the study (Table 1). Of them, 23 (37%) received t-PA treatment before EVT. All the patients had severe stenosis/occlusion of a unilateral side of MCA (*n* = 41) or internal carotid (*n* = 21). We also recruited 77 healthy volunteers as controls (Table 1). There were no significant differences in gender, age, BMI and TG between the AIS patients and controls. However, AIS patients were associated with higher systolic blood pressure (SBP), diastolic blood pressure (DBP), fast blood glucose, heart rate and C-Reactive protein (CRP) compared with healthy controls (all *p* < 0.05).

**Table 1:** The demographic and clinical characteristics of healthy controls and patients

Variable	Healthy controls (***n*** = 77)	Acute ischemic stroke patients (***n*** = 62)	***P***-value
Gender (male/female)	53/24	45/17	0.710
Age (years)	55.75 ± 11.32	55.61 ± 14.53	0.949
SBP (mmHg) on admission	119.54 ± 13.12	137.85 ± 22.15	< 0.001^*^
DBP (mmHg) on admission	72.22 ± 10.62	77.43 ± 16.60	0.031^*^
Fast blood glucose (mmol/L)	5.82 ± 1.55	7.29 ± 2.40	< 0.001^*^
Heart rate (bpm)	68.09 ± 8.71	75.71 ± 17.82	0.038^*^
BMI	24.03 ± 2.65	23.49 ± 3.19	0.288
TG (mmol/L)	1.05 ± 0.28	1.24 ± 0.67	0.083
CRP (mg/L)	0.60 (0.33,1.20)	7.89 (2.19, 20.47)	< 0.001^*^
Smoker	0 (0)	8 (38.10)	–
Drinker	0 (0)	4 (19.05)	–
Hypertension	0 (0)	8 (38.10)	–
Diabetes mellitus	0 (0)	2 (9.52)	–

Values are expressed as mean ± SD or numbers (%) or median (inter- quartile range, IQR)

*SBP* Systolic blood pressure, *DBP* Diastolic blood pressure, *TG* Triglyceride, *BMI* Body Mass Index, *CRP* Hypersensitive C-Reactive Protein, *NA* Not applicable

*: significant difference in comparing with control

*P*-value: *p*-value of comparing between healthy controls and patients

### Dynamic CA in controls and AIS patients

Dynamic CA was assessed at 24 h, 72 h, and 7 d after stroke onset in all AIS patients, but only once in healthy controls. Compared with healthy controls, PD values on bilateral hemispheres were significantly lower in AIS patients at different time points (Table 2). In AIS patients, PD values on the ipsilateral side were significantly lower than that on the contralateral side at 24 h and 7 d after onset. At 72 h, there was a trend towards lower values of PD on the ipsilateral side compared with the contralateral side, although no statistical significance was reached (*p* = 0.066) (Table 2)**.** Univariate linear regression analysis showed that fast blood glucose on admission was associated with PD on the ipsilateral hemisphere at 24 h after symptom onset, β = − 4.453, *p* = 0.009.

**Table 2:** The cerebral autoregulation value in controls and patients

Variable	Healthy controls	AIS Patients		***P***-value
		contralateral side	ipsilateral side	
Phase on 24 h after onset	62.00 ± 18.43	40.50 ± 28.11^a^	29.07 ± 25.71^a^	0.012^#^
Phase on 72 h after onset	62.00 ± 18.43	31.71 ± 23.88^a^	26.37 ± 21.86^a^	0.066
Phase on 7d after onset	62.00 ± 18.43	37.08 ± 27.06^a^	29.13 ± 22.37^a^	0.020^#^

^a^significant difference in comparing between controls and patients

^#^significant difference in comparing the dynamic cerebral autoregulation value between the contralateral side and the ipsilateral side

*P*: *P*-value of comparing between the contralateral side and the ipsilateral side

### Dynamic CA in AIS patients with favourable and unfavourable outcomes

During follow up, 21 (34%) patients had favourable outcome and 41 (66%) patients had unfavourable outcome at 90 days after stroke onset (Table 3). Unfavourable outcome patients were more likely to have higher NIHSS, fast blood glucose, BMI, TG and CRP compared with those with favourable outcome (all *p* < 0.05). However, the ratio of t-PA treatment and ICA stenosis/occlusion, duration from symptoms onset to hospital and the door to needle time (DNT) were all comparable between the 90-day favourable and unfavourable outcome groups (all *p* > 0.05).

**Table 3:** The demographic and clinical characteristics of patients with 90-day favourable outcome (mRS of 0–2) and unfavourable outcome (mRS of 3–6)

Variable	Favourable outcome (***n*** = 21)	Unfavourable outcome (***n*** = 41)	***P***-value
Gender (male/female)	17/4	28/13	0.375
Age (years)	51.48 ± 11.23	57.73 ± 15.67	0.109
SBP (mmHg) on admission	134.10 ± 20.69	139.73 ± 22.87	0.358
DBP (mmHg) on admission	78.05 ± 16.28	77.13 ± 15.45	0.831
Fast blood glucose (mmol/L)	6.06 ± 1.88	7.92 ± 2.41	0.003*
Heart rate (bpm)	74.38 ± 14.50	75.71 ± 17.82	0.792
BMI	22.18 ± 3.14	24.16 ± 3.03	0.019*
TG (mmol/L)	0.98 ± 0.30	1.37 ± 0.75	0.018*
CRP (mg/L)	3.70 (1.15, 7.13)	10.96 (4.57, 26.00)	0.003*
Smoker	8 (38.10)	19 (46.34)	0.596
Drinker	4 (19.05)	13 (31.71)	0.375
Hypertension	8 (38.10)	18 (43.90)	0.788
Diabetes mellitus	2 (9.52)	11 (26.83)	0.187
NIHSS on admission	9 (5, 14)	15 (11, 20)	< 0.001*
Large artery stenosis/occlusion			0.777
MCA	13 (61.90)	28 (68.29)	
ICA	8 (38.10)	13 (31.71)	
t-PA treatment	9 (42.86)	14 (34.15)	0.583
Onset to hospital time (min)	348.88 ± 280.57	288.33 ± 180.33	0.372
DNT (min)	115.06 ± 49.05	95.53 ± 58.58	0.258
The time intervals between completed EVT and dCA measurement (hour)	9.33 ± 3.57	7.76 ± 3.75	0.117

Values are expressed as mean ± SD or numbers (%) or median (inter- quartile range, IQR)

*SBP* Systolic blood pressure, *DBP* Diastolic blood pressure, *TG* Triglyceride, *BMI* Body Mass Index, *CRP* Hypersensitive C-Reactive Protein, *NIHSS* National Institutes of Health Stroke Scale, *MCA* Middle cerebral artery, *ICA* Internal carotid artery, *DNT* Door to needle time, *dCA* dynamic cerebral autoregulation, *EVT* Endovascular therapy

^*^significant difference in comparing between favourable-outcome group and unfavourable-outcome group

*P*-value: *p*-value of comparing between favourable-outcome group and unfavourable-outcome group

The dCA values in the favourable outcome and unfavourable outcome patients were presented in Table 4. On the contralateral side, the PD value showed no significant difference between these two groups at different time-points (all *p* > 0.05). On the ipsilateral side, however, the PD value at 24 h and 72 h were significantly lower in patients with unfavourable outcome than those with favourable outcome (all *p* < 0.05). Conversely, no significant differences were observed in 7d after onset.

**Table 4:** The dynamic cerebral autoregulation value in favourable outcome and unfavourable outcome patients

Variable	Favourable outcome (***n*** = 21)	Unfavourable outcome (***n*** = 41)	***P***-value
Phase on 24 h after onset			
Phase on contralateral side	51.97 ± 33.58	34.92 ± 23.59^a^	0.072
Phase on ipsilateral side	45.47 ± 27.67^a^	20.64 ± 20.32^a^	0.001^#^
Phase on 72 h after onset			
Phase on contralateral side	39.97 ± 28.35^a^	26.20 ± 18.93^a^	0.066
Phase on ipsilateral side	34.70 ± 21.06^a^	21.09 ± 21.01^a^	0.032^#^
Phase on 7d after onset			
Phase on contralateral side	45.59 ± 28.65^a^	31.23 ± 25.14^a^	0.180
Phase on ipsilateral side	39.74 ± 24.57^a^	22.49 ± 18.71^a^	0.054

^a^significant difference in comparing with control after adjusting confounding factors

#: significant difference in comparing between favourable-outcome group and unfavourable-outcome group

*P*: *P*-value of comparing between favourable-outcome group and unfavourable-outcome group

**Fig. 1:**
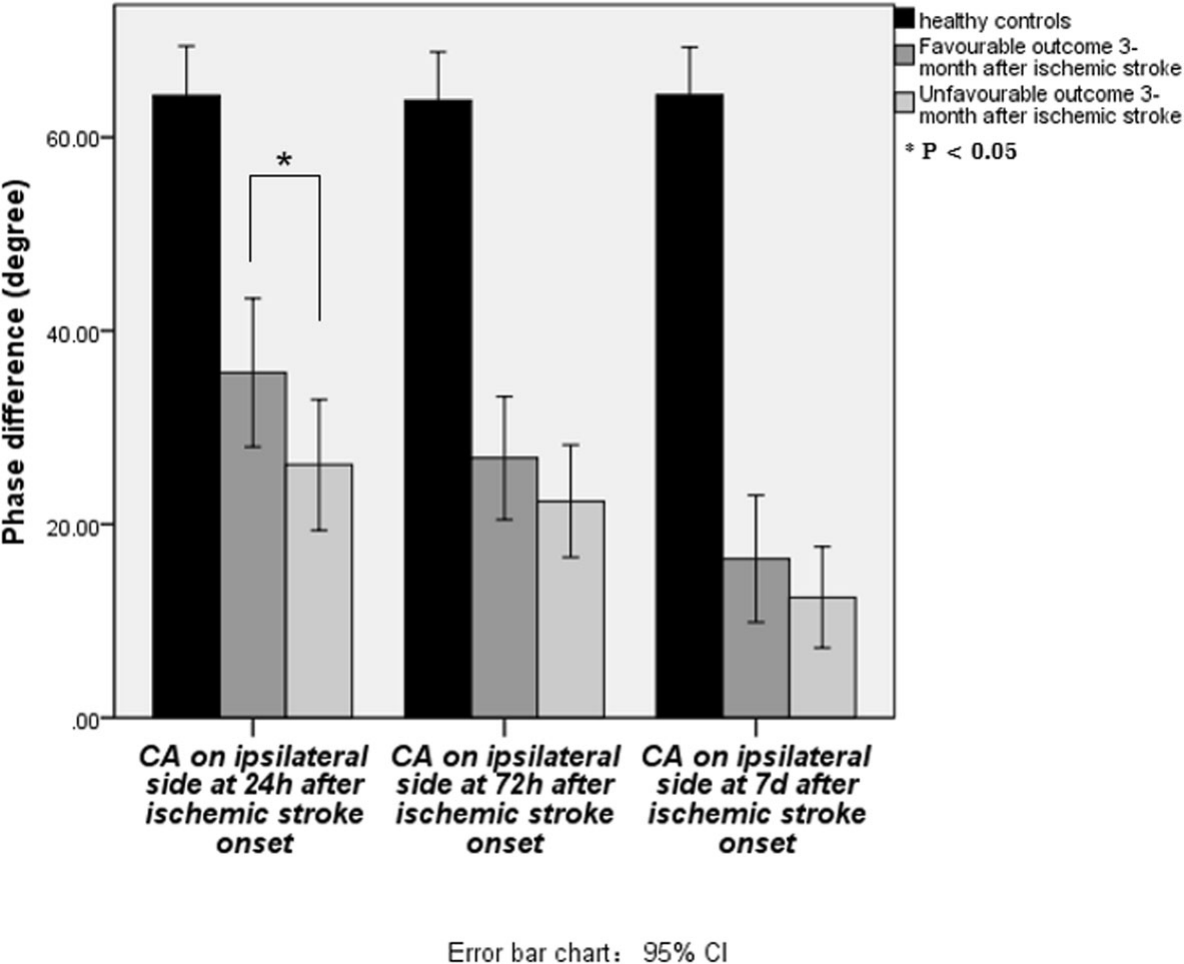
On the ipsilateral side, however, the PD value at 24 h was significantly lower in patients with unfavourable outcome than those with favourable outcome

**Fig. 2:**
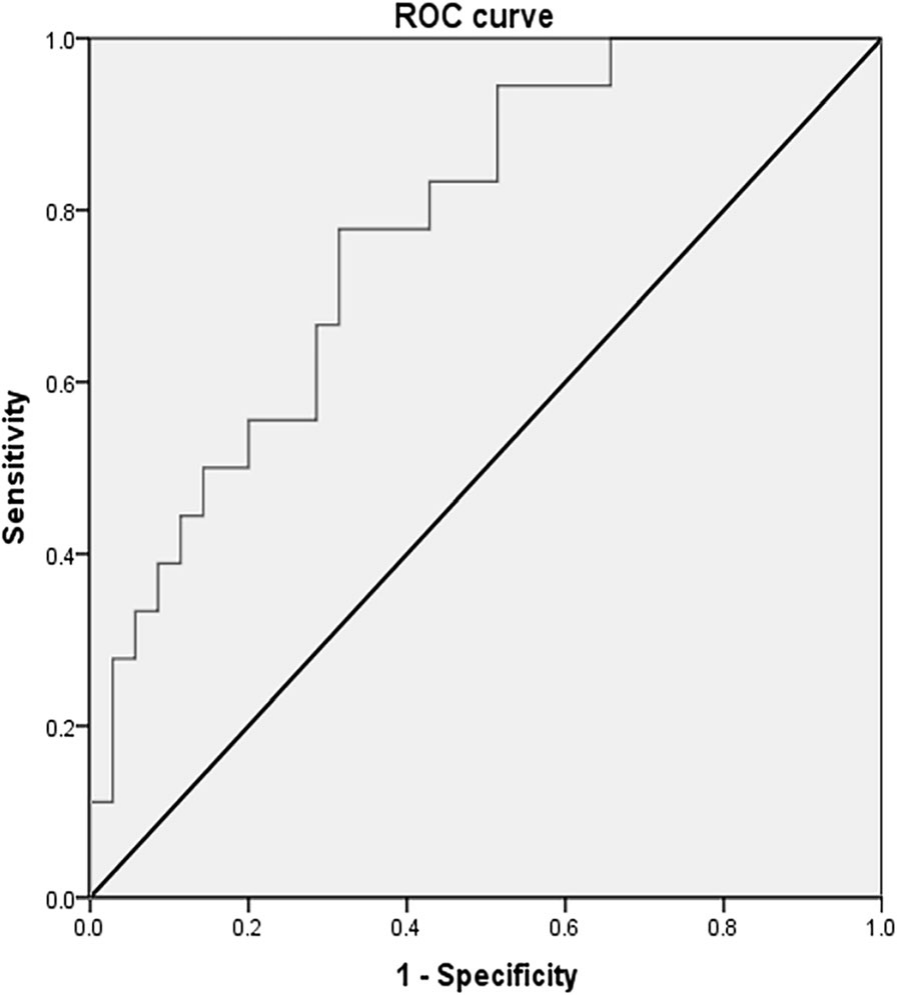
The ROC curve the optimal cutoff value of the ipsilateral PD for predicting unfavourable outcomes was < 26.93° (sensitivity 76.50%, specificity 69.20%)

After adjusting the confounding factors including fast blood glucose, triglyceride (TG), body mass index (BMI), C-reactive protein (CRP), and NIHSS on admission, logistic regression analysis showed that PD value on the ipsilateral hemisphere at 24 h after onset was an independent predictor of clinical outcome, (adjusted OR = 0.889, 95% CI: 0.813–0.971, *P* = 0.009) (Table 5, Fig. 1). Lower ipsilateral PD value at 24 h after onset was correlated with higher mRS (*r* = − 0.402, *P* = 0.007), indicating poorer clinical outcomes. The ROC curve was performed to determine the cutoff point that optimized the sensitivity and specificity associated with clinical outcomes. The optimal cutoff value of the ipsilateral PD for predicting unfavourable outcomes was < 26.93° (sensitivity 76.50%, specificity 69.20%) (Fig. 2). The area under the curve of the ROC curve was 0.781.

**Table 5:** Multivariate analysis of clinical characteristics and dynamic cerebral autoregulation for favorable long-term outcome

Variables	Crude OR (95% CI)	***P***-value	Adjusted OR (95% CI)	***P***-value
**Clinical factors**				
Fast blood glucose (mmol/L)	1.726 (1.171, 2.544)	0.006*	3.523 (1.255, 9.885)	0.017*
BMI	1.250 (1.027, 1.521)	0.026*		0.623
TG (mmol/L)	3.401 (0.937, 12.343)	0.063		0.760
CRP (mg/L)	1.088 (1.008,0.175)	0.031*		0.162
NIHSS on admission	1.276 (1.105, 1.474)	0.001*	1.825 (1.155, 2.886)	0.010*
**Cerebral autoregulation**				
dCA on 24 h after onset				
Phase on ipsilateral side	0.955 (0.926, 0.985)	0.004*	0.889 (0.813, 0.971)	0.009*
Phase on contralateral side	0.978 (0.956, 1.000)	0.048*		0.184
dCA on 72 h after onset				
Phase on ipsilateral side	0.970 (0.942, 0.998)	0.039*		0.205
Phase on contralateral side	0.974 (0.948, 1.000)	0.053		0.183
dCA on 7d after onset				
Phase on ipsilateral side	0.962 (0.923, 1.003)	0.069		0.259
Phase on contralateral side	0.979 (0.950, 1.010)	0.180		

*TG* Triglyceride, *BMI* Body Mass Index, *CRP* Hypersensitive C-Reactive Protein, *NIHSS* National Institutes of Health Stroke Scale

^*^significant difference in comparing between favourable-outcome group and unfavourable-outcome group, *p* < 0.05

## Discussion

Endovascular therapy is increasingly used to treat patients with occlusions of the large intracranial arteries. However, the effect factors on clinical outcome after EVT are still unclear. In the study, we found that dCA was impaired bilaterally over the first 7 days after symptom onset, even those patients receiving EVT. The impairment of dCA on the ipsilateral side at 24 h after onset was associated with clinical outcome in AIS patients who received EVT. We also determined the optimal cutoff value of the dCA index for favourabe 90-day outcome prediction.

In the study, we enrolled patients who received EVT with or without t-PA treatment previously. We found similar safety outcomes and no significant difference in functional independence with EVT after intravenous t-PA, as compared with without t-PA treatment. That may be attributed to that we determine EVT patients according to the NIHSS score, MRA, and DWI-fluid attenuated inversion recovery mismatch. Further large sample size research is needed.

### Choosing dCA as the marker

Recently, numerous studies emphasize the significant predictive value of cerebrovascular reserve in ischemic stroke [21, 22]. Reduced cerebrovascular reserve indicates that the arteriolar vasodilation activity is not able to maintain stable blood flow properly in the region of cerebral hypoperfusion [21, 23, 24]. DCA is the pivot pathophysiological process to maintain stable cerebrovascular reserve capacity. It was reported that impaired dCA might trigger ischemic brain lesions [14, 25]. The underlying pathophysiological mechanisms might be that the severe stenosis may significantly reduce cerebral perfusion pressure, which could reach its lower limit for autoregulation. Under these conditions, even slight reductions of BP may result in inadequate cerebral perfusion and would not be able to protect the brain from hemodynamic ischemic events [26]. With the development of TCD monitoring, dCA can be evaluated by simultaneous assessment of spontaneous fluctuations in BP and BFV in MCA (assessed by TCD) using different testing modalities in the time and frequency domain [20, 27]. The methodology of such an approach based on the concept that cerebral autoregulatory system functions as a high-pass filter, which means that high-frequency fluctuations of BP are normally passing through BFV unimpededly while low-frequency oscillations are dampened. As a result, slow waves of BFV do not occur simultaneously to similar waves of BP but with a time delay. A PD of 0°indicates total absence of autoregulation, while a large positive PD of 40°-70°can be regarded as intact autoregulation. In our study, the PD obtained from oscillations in ABP and CBFV for MCA in healthy subjects were similar, which is consistent with previous studies. Comparing with healthy controls, we found that dCA on both the ipsilateral hemisphere and the contralateral hemisphere were impaired in AIS patients. Consistently with our study, previous researches also reported bilateral impaired CA in the acute stroke [28, 29]. The mechanism of this trans-hemispheric communication may be diaschisis where there is distant functional depression due to the effects of loss of axons (mainly facilitatory) arising at the site of the lesion and, in the case of the cerebral hemispheres, these may synapse with neurons in the contralateral hemisphere via the corpus callosum [30]. However, further basic research was needed to confirm it. In addition, Dawson et al., have indicated that dCA appeared impaired bilaterally and remained so for at least 1 to 2 weeks over the subacute post-stroke period [31]. In a follow-up study, CA was also abnormal on the affected side > 2 months after stroke onset [32]. All those were closely in keeping with our finding, that the dysautoregulation still existed 7 days after symptom onset, even receiving EVT. That means our results support the theory of an additional secondary autoregulatory failure in acute cerebral ischemia. However, due to time restriction of EVT, we could not assess preoperative dCA, we still do not understand the detail change of postoperative dCA through comparing with preoperative dCA.

### Influence factors on clinical outcome after EVT

In our study, we observed significantly lower dCA on the ipsilateral side at 24 h after symptom onset in unfavourable-outcome patients than favourable-outcome patients even after adjusting confounding factors. The t-test showed contralateral dCA with a difference bordering on significance between favourable-outcome and unfavourable-outcome groups. However after adjusting confounding factors, no significance of contralateral dCA was found. Larger sample size research is needed to explore it in future. The ROC showed that the PD value on the ipsilateral side at 24 h lower than 26.93° indicated unfavourable 90-day-outcome in patients who received EVT. In the studies about AIS patients performed by Chi et al. [33] and Castro el al [34], PD values were associated with mRS score > 2 at 3 months in patients with moderate to severe stroke even in the multivariate analysis. Castro et al. suggested PD < 37° as a cutoff to predict unfavourable outcomes, whereas Chi et al. suggested a cutoff of PD < 61°. The difference may be because of the different frequency band selection in transfer function analysis (TFA), that Chi et al. analyze PD in very low frequency. Besides, we noticed that our cut-off PD value was lower than the value reported by previous research. That may indicate that EVT may improve the tolerance to hemodynamic fluctuations. Saur et al. [35] and Reinhard et al. [25] suggested that the development of cerebral dysautoregulation may be particularly critical during the first days of reperfusion in larger infarctions. The first day is probably also the most critical period with regard to functional brain reorganization. It might be explained that in the acute stage after EVT, hemodynamic status changed. Focal dysautoregulation in the ischemic core and the former penumbral area might enhance the reperfusion injury and lead to secondary endothelial dysfunction, losing the protective function for the ischemic penumbra. Our findings indicate that special attention should be paid to the early acute stage of large acute ischemic stroke when secondary autoregulatory failure can evolve mainly in the affected vascular territory. In patients treated with EVT, BP control strategies in large ischemic stroke should be guided by the status of vessel recanalization and autoregulatory capacity, especially at 24 h after ischemic stroke onset. Consistent with our finding although with a different dCA monitoring measurement, a recent research suggested that continuous estimation of autoregulation-based treatment strategies after EVT was feasible and could provide a BP range for individual patients tailored to their own physiology [22]. In future, larger sample size research is needed to observe dCA status when changing BP level.

The present analysis has some limitations. Firstly, we recruited more male subjects than female in our study, since there are more male stroke patients than female in China [36], and female subjects are more likely to have insufficient bilateral temporal bone windows for insonation due to the low density of the temporal bone [37]. Secondly, the sample size of our research is small, further larger sample size research is needed. Thirdly, in our study, we used heparin during EVT, which might be the limitation of the protocol. Further relative research is needed.

## Conclusion

In conclusion, dCA are impaired on the ipsilateral hemisphere and contralateral hemisphere during the first 7 days after AIS symptom onset. Dynamic CA on the ipsilateral hemisphere at 24 h after symptom onset is independently associated with clinical outcomes in AIS patients who received EVT. Impaired dCA can be set up as an early hemodynamic marker to guide acute stage management in patients who received EVT. It provides a novel prognostic tool to improve clinical outcomes achieving personalized treatment.

## Data Availability

The raw/processed data required to reproduce these findings cannot be shared at this time as the data also forms part of an ongoing study.

## Abbreviations

EVT: Endovascular therapy
AIS: Acute ischemic stroke
dCA: Dynamic cerebral autoregulation
MCA: Middle cerebral artery
PD: Phase difference
mRS: Modified Rankin Scale
ROC: Receiver operation characteristics
CBF: Cerebral blood flow
ABP: Arterial blood pressure
NIHSS: National Institutes of Health Stroke Scale
TICI: Thrombolysis In Cerebral Infarction
MRA: Magnetic resonance angiography
ASL: Arterial Spin Labeling
TCD: Transcranial doppler sonography
CD: Carotid artery color doppler
IQR: Interquartile range
TG: Triglyceride
BMI: Body mass index
CRP: C-reactive protein
SBP: Systolic blood pressure
DBP: Diastolic blood pressure
TFA: Transfer function analysis
